# Optimizing self-expandable metallic stent placement for malignant ileocecal obstruction: Role of pre-stenting contrast enema and device selection

**DOI:** 10.1055/a-2733-0780

**Published:** 2025-11-10

**Authors:** Akihiro Maruyama, Hiroshi Nakayabu, Hirotaka Takeshima, Hiroki Kato, Shintaro Tominaga, Makoto Kobayashi

**Affiliations:** 1 37036 Gastroenterology, Yokkaichi Municipal Hospital, Yokkaichi, Japan

**Keywords:** Endoscopy Lower GI Tract, Stenting, Colorectal cancer, Quality and logistical aspects, Preparation

## Abstract

**Background and study aims:**

Self-expandable metallic stent (SEMS) placement is an established intervention for malignant colorectal obstruction, but the ileocecal region presents unique anatomical challenges. This study aimed to evaluate the technical success of SEMS placement for malignant ileocecal obstruction and to examine the impact of pre-stenting preparation and device selection.

**Patients and methods:**

We retrospectively analyzed 72 patients with right-sided malignant colonic obstruction (13 ileocecal, 59 other segments) treated between January 2011 and March 2025. The primary outcome was the technical success rate; procedure efficiency was the secondary outcome. Clinical success was also evaluated in the ileocecal group according to treatment intent. All ileocecal cases underwent contrast liquid enema-assisted navigation (CLEAN) to assist device selection. Subgroup analyses examined scope diameter, hood shape, operator experience, and catheter tip mobility.

**Results:**

Technical success was significantly lower in the ileocecal group (76.9% vs. 98.3%, *P* = 0.017), whereas median procedure times were similar (40.0 vs. 35.0 minutes, *P* = 0.934). In the ileocecal group, all patients with technical success also achieved clinical success. No major complications occurred. Tapered hoods significantly shortened procedure time (26.0 vs. 50.0 minutes, *P* = 0.018), and expert operators completed procedures faster than trainees (30.5 vs. 58.0 minutes, *P* = 0.042). Although movable-tip catheters and smaller-diameter scopes showed no statistical differences, selected cases demonstrated procedure advantages.

**Conclusions:**

SEMS placement in the ileocecal region is technically more challenging than in other right-sided segments. Procedure optimization – potentially aided by CLEAN, tapered hoods, and experienced operators—may help overcome anatomical difficulties while maintaining safety.

## Introduction

Colorectal cancer is the third most common cancer worldwide, with 10% to 20% of patients presenting with gastrointestinal obstruction 1 . Malignant colorectal obstruction is associated with severe complications such as electrolyte imbalance and bowel perforation, necessitating urgent decompression. Since the early 1990s, following the report by Dohmoto et al 2 about use of SEMSs (self-expanding metal stents), endoscopic SEMS placement has been widely performed for malignant obstruction in the left colon. However, in the right colon, technical challenges such as poor visualization due to residual stool and bowel tortuosity have often led to surgical intervention being prioritized 3 . In recent years, advance in devices, including catheters with movable tips and stents with reduced axial force that minimize risk of perforation, have increased the feasibility of SEMS placement in the right colon 4 .

SEMS placement can be classified into two main purposes: bridge to surgery (BTS) and palliation (PAL). BTS avoids the need for colostomy and facilitates minimally invasive laparoscopic surgery. In contrast, PAL aims to relieve obstruction, shorten hospitalization duration, and expedite initiation of chemotherapy. Both approaches enable early resumption of oral intake, contributing significantly to improvement in patient quality of life (QoL) 5 . Therefore, when a SEMS can be placed safely and effectively, it becomes an invaluable therapeutic option.

The ileocecal region, however, poses unique anatomical challenges. Presence of the ileocecal valve and the angulated connection with the small intestine are notable features. Furthermore, its distance from the anus increases the technical difficulty of SEMS placement in this area. Although case reports have documented successful outcomes using catheters with movable tips or stents with reduced axial force, comprehensive studies remain limited 6 7 8 . In this study, we retrospectively analyzed safety and efficacy of SEMS placement for malignant ileocecal obstruction and examined the effectiveness of preparation and device selection in overcoming the associated technical challenges.

## Patients and methods

### Study design

This retrospective single-center study included 72 patients treated between January 2011 and March 2025. Patients were eligible if they had malignant obstruction confirmed by physical and imaging findings, with lesions located in the ileocecal region (13 cases) or other right-sided colonic segments, including the ascending and transverse colon (59 cases). All patients were diagnosed with adenocarcinoma through biopsy or surgical specimens. Patients who were not considered suitable for stent placement due to complications such as obstructive colitis, perforation, or abscess formation were excluded from the analysis. The study was approved by the Ethics Committee of Yokkaichi Municipal Hospital and conducted in accordance with the Declaration of Helsinki.

### Statistical analysis

Comparisons of technical success rates between the ileocecal region and other right-sided colonic segments were conducted using Fisher’s exact test. For analysis of procedure duration, the Mann-Whitney U test was performed due to the small sample size and potential non-parametric distribution of data.

Subgroup analyses were conducted to investigate factors influencing technical success and procedure efficiency in the ileocecal region. These subgroup factors were specifically chosen because this study focused on device selection, and operator expertise was also considered influential in device selection and procedure outcomes.

The following factors were examined: scope tip diameter (≤ 10 mm vs. > 10 mm), hood shape (tapering vs. non-tapering), operator experience (expert vs. trainee), and catheter type (movable-tip vs. non-movable-tip). Fisher’s exact test was used to compare categorical variables and the Mann-Whitney U test was applied for comparisons of procedure times due to the limited sample size and non-parametric data distribution.

All statistical tests were performed using Python libraries, including SciPy and Statsmodels, and *P* < 0.05 was considered statistically significant. All confidence intervals (CIs) are presented at the 95% confidence level.

### SEMS placement procedure

Endoscopic stent placement was performed by two physicians in all cases, with one serving as the operator and the other as the assistant. Physicians with experience in more than 20 cases of colorectal stent placement were classified as experts, whereas those with fewer than 20 cases were considered trainees. Procedures conducted by trainees were supervised by an expert.

**Fig. 1:**
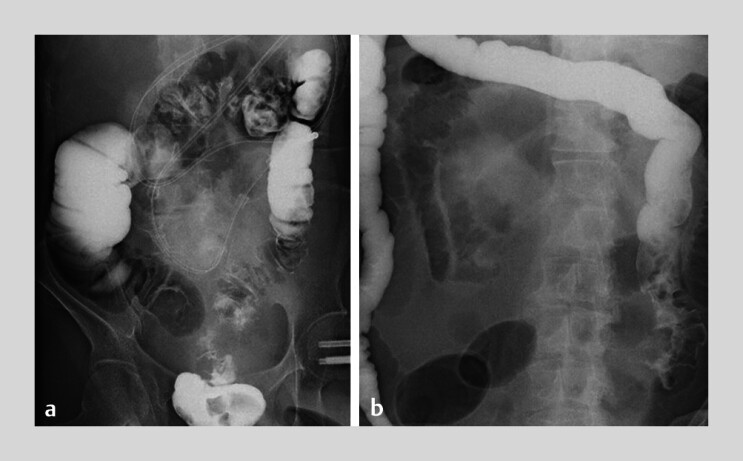
Fluoroscopic gastrografin enema images in patients with malignant ileocecal obstruction. **a** Case 8: Supine position. The ileocecal region is visualized using Gastrografin contrast under fluoroscopy. **b** Case 11: Prone position. The anatomical alignment and stricture site are evaluated using Gastrografin contrast under fluoroscopy.

**Fig. 2:**
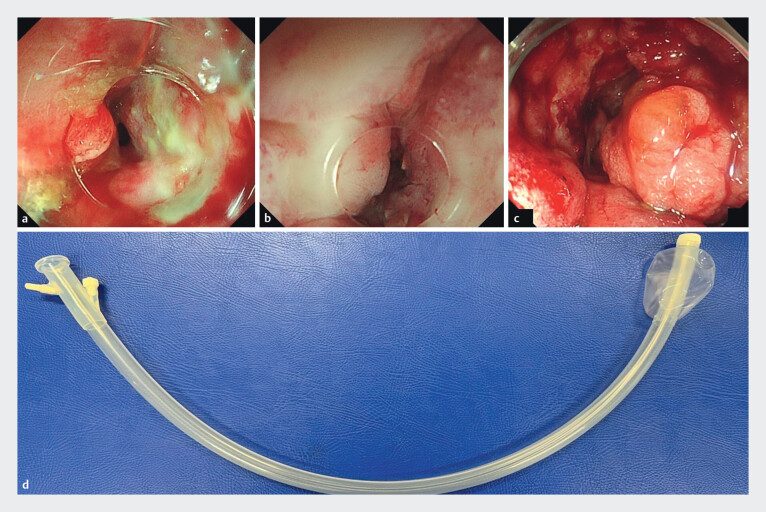
Endoscopic appearance of different hoods used during SEMS placement for malignant ileocecal obstruction. **a** ST-short hood applied and pressed against the stricture site (Case 10). **b** Cast hood applied and pressed against the stricture site (Case 11). **c** Conventional attachment hood applied and pressed against the stricture site (Case 4). **d** Balloon-equipped overtube (ST-CB1) used to stabilize the endoscope and facilitate approach to the lesion.

Bowel preparation for all ileocecal cases was achieved using a Gastrografin enema, a technique we defined as the contrast liquid enema-assisted navigation (CLEAN) method ( Fig. 1 ). Bowel preparation quality for ileocecal cases was assessed using the Boston Bowel Preparation Scale (BBPS) at time of SEMS placement, and total scores (range 0–9) were recorded. Following contrast enema, device selection—including type of endoscope, catheter, and hood—was determined by the operator based on anatomical findings and stricture characteristics. After preparation, an endoscope (CF-H260AI, pCFH290AI, CF-HQ290I, or JF-260V; Olympus, Tokyo, Japan) equipped with a disposable attachment (Olympus, Tokyo, Japan), ST short attachment (FUJIFILM, Tokyo, Japan), or cast attachment (TOP, Tokyo, Japan) was advanced to the ileocecal region. For cases in which the bowel was highly flexible, a balloon-assisted overtube ST-CB1 (Olympus, Tokyo, Japan) was used ( Fig. 2 ). Contrast medium was injected into the stricture for evaluation.

**Fig. 3:**
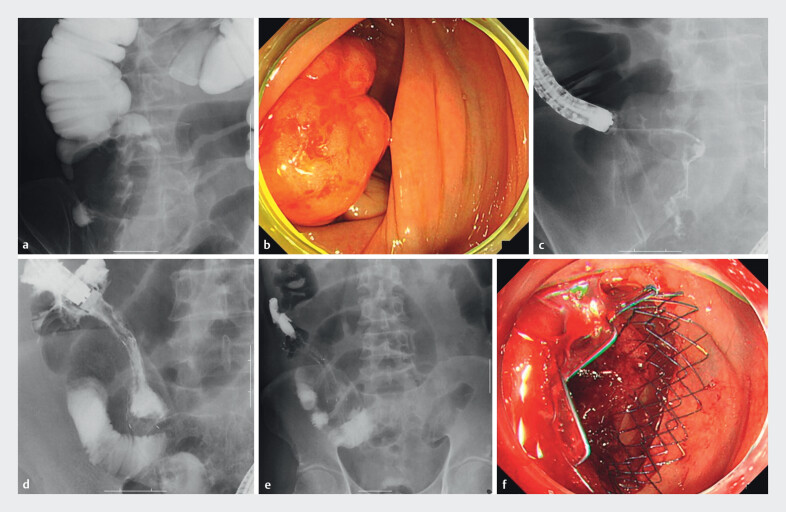
Representative images of SEMS placement procedure for malignant ileocecal obstruction. **a** Fluoroscopic image of Gastrografin enema performed prior to endoscopic insertion, clearly delineating the obstruction site. **b** Endoscopic visualization of the obstructive tumor prior to stent placement. **c** Fluoroscopic image capturing the moment when the guidewire and catheter successfully traverse the obstructive lesion. **d** Fluoroscopic confirmation of contrast medium passing through the stent immediately after deployment, verifying successful relief of obstruction. **e** Post-procedure abdominal radiograph showing optimal stent placement and expansion. **f** Endoscopic appearance immediately following successful SEMS deployment, confirming accurate stent positioning.

The stricture was traversed using a guidewire (Revo Wave Standard or Revo Wave Hard; PIOLAX, Kanagawa, Japan). Following this, a catheter (tandem XL; Boston Scientific, Marlborough, MA, USA; Seekingtome zero; ABIS, Hyogo, Japan; or PR-233Q; Olympus, Tokyo, Japan) was advanced, and imaging proximal to the stricture was performed. Based on the findings, a stent was selected and deployed: either Natur fit (Boston Scientific, Marlborough, Massachusetts, United States), Niti-S (Taewoong Medical, Kimpo, Korea), or WallFlex (Boston Scientific, Marlborough, Massachusetts, United States) ( Fig. 3 ). Device selection, including the endoscope, catheter, stent, and attachment, was determined by the physician according to the clinical scenario. Details of the devices used and pre-procedure strategies implemented in this study are summarized in Table 1 .

**Table:** **Table 1** Device and procedure details for 13 cases of ileocecal stenting.

**No.**	**Age**	**Sex**	**Scope**	**Hood**	**Catheter**	**Stent**	**ST**	**TS**	**CS**	**Time (min)**	**OP**	**AS**	**BBPS**
Case 1	78	M	CF-H260AI	Attachment	PR-233Q	NitiS 12 cm	○	○	○	61	Expert	Trainee	3
Case 2	76	M	CF-H260AI	Attachment	TandemXL	NitiS 6 cm	○	○	○	41	Expert	Trainee	3
Case 3	57	M	CF-H260AI	Attachment	TandemXL•PR-233Q	Natur fit 9 cm	○	○	○	61	Trainee	Expert	2
Case 4	76	M	CF-HQ290I	Attachment	PR-233Q	Natur fit 9 cm	○	×	×	58	Trainee	Expert	4
Case 5	47	F	pCF-H290TI	Attachment	TandemXL	Natur fit 9 cm	○	○	○	33	Expert	Trainee	4
Case 6	79	F	pCF-H290TI	ST-short	TandemXL	Natur fit 9 cm	○	○	○	25	Expert	Expert	4
Case 7	71	F	CF-HQ290TI	ST-short	TandemXL	Natur fit 9 cm	○	○	○	28	Expert	Expert	3
Case 8	65	M	pCF-H290TI→JF-260V	None/ST-CB1	Seekingtome	Natur fit 9 cm	○	○	○	50	Expert	Trainee	3
Case 9	72	M	pCF-H290TI	ST-short	Seekingtome	none	×	×	×	23	Expert	Trainee	4
Case 10	79	F	pCF-H290TI	ST-short	Seekingtome	Natur fit 9 cm	○	○	○	19	Expert	Expert	4
Case 11	53	M	pCF-H290TI	Cast	Seekingtome	Natur fit 9 cm	○	○	○	51	Trainee	Expert	5
Case 12	75	F	CF-HQ290I	Attachment	TandemXL•PR-233Q	none	×	×	×	50	Expert	Expert	4
Case 13	77	M	CF-HQ290I	ST-short	TandemXL	Natur fit 9cm	○	○	○	27	Expert	Expert	4
AS, assistant; BBPS, Boston Bowel Preparation Scale; CS, clinical success; success; OP, operator; ST, stricture transverse; TS, technical.													

### Outcome measurement

The primary outcome of this study was the technical success rate, defined as successful placement of the SEMS across the stricture with appropriate expansion, confirmed by fluoroscopic and endoscopic findings. A procedure was considered technically successful when the guidewire and catheter successfully passed the obstruction and the stent was fully deployed without migration or malposition. Clinical success also was evaluated in the ileocecal group according to treatment intent. In patients treated with BTS, it was defined as absence of complications, including re-obstruction, stent migration, and abscess formation, thereby enabling subsequent surgical intervention as planned. In cases treated with PAL intent, it was determined by symptomatic relief sufficient to allow discharge without need for additional intervention. The secondary outcome was procedure efficiency, measured as total procedure time from endoscope insertion to successful stent deployment. This was assessed across different colonic segments (ileocecal region, ascending colon, transverse colon) and subgroups based on operator experience, catheter type, endoscopic attachment, and scope diameter.

## Results

### Patient characteristics

A total of 72 patients with right colon obstruction were included. Mean age was 74.4 years (±13.1), with a male-to-female ratio of 41:31. Among these, 47 patients underwent stent placement for BTS purposes, whereas 25 were treated for palliation ( Table 2 ).

**Table:** **Table 2** Baseline characteristics of patients undergoing SEMS placement for right-sided colonic obstruction.

**Variable**	**Ileocecal**	**Ascending**	**Transverse**	**Total**
**Total (n)**	**13**	**20**	**39**	**72**
Mean age (years)	69.6 ± 10.8	80.3 ± 10.2	72.9 ± 14.2	74.4 ± 13.1
Male/female (n)	8/5	15/5	18/21	41/31
Stage II/III/IV (n)	3/2/8	1/7/12	13/12/14	17/21/34
BTS/PAL (n)	8/5	12/8	27/12	47/25
BTS, bridge to surgery; PAL, palliation; SEMS, self-expanding metal stent.				

### Technical and clinical outcomes

The technical success rate was 76.9% (10/13; 95% CI 49.7%-91.8%) in the ileocecal region and 98.3% (58/59; 95% CI 91.0–99.7%) in the other right-sided colonic segments ( *P* = 0.017). In the ileocecal group, all patients with technical success also achieved clinical success. Median procedure duration was 40.0 minutes (interquartile range [IQR] 30–56 minutes) in the ileocecal region, compared with 35.0 minutes (IQR 26–52 minutes) in the other right-sided colonic segments, with no significant difference observed between groups ( *P* = 0.934). No major complications, such as perforation, significant bleeding, or infection, were observed ( Table 3 ). The dataset did not provide detailed information on minor complications. All three patients who experienced technical failure subsequently underwent surgical intervention. In the BTS group, no adverse events occurred during the preoperative period. In contrast, in the PAL group, follow-up after discharge was incomplete in several cases due to transfers to other facilities and other logistical reasons, which limited assessment of delayed complications.

**Table:** **Table 3** Technical and clinical outcomes of SEMS placement in the ileocecal region and other right-sided colonic segments.

	**Ileocecal region (N = 13)**	**Other right-sided colonic segments (N = 59)**
Technical success	10/13	58/59
Success rate (%) (95% CI)	76.9% (49.7%-91.8%)	98.3% (91.0%-99.7%)
Procedure time (min)	40.0 (30–56)	35.0 (26–52)
Stent Jumping	1/13 (7.7%)	1/59 (1.7%)
Perforation	0/13 (0.0%)	0/59 (0.0%)
Bleeding	0/13 (0.0%)	0/59 (0.0%)
SEMS, self-expanding metal stent.		

### Subgroup analysis

Subgroup analyses were conducted to evaluate factors influencing outcomes of SEMS placement for malignant ileocecal obstruction ( Table 4 ). The primary objective was to identify key determinants of technical and clinical success as well as procedure efficiency, aiming to optimize treatment strategies. These analyses focused on four aspects: scope diameter (≤ 10 mm vs. > 10 mm), endoscopic attachment shape (tapering vs non-tapering), operator experience (expert vs trainee), and type of catheter used (movable vs non-movable tip).

**Table:** **Table 4** Subgroup analysis of factors associated with technical success and procedure time in ileocecal SEMS placement.

**Subgroup**	**Success rate (%) (95% CI)**	**Procedure time (min)**	***P* value (procedure time) **
Scope tip diameter (≤ 10 mm vs. > 10 mm)	83.3% (43.6%-97.0%) vs. 71.4% (35.9%-91.8%)	29.0 (23.5–45.8) vs. 50.0 (34.5–59.5)	0.115
Hood type (tapering vs. non-tapering)	83.3% (43.6%-97.0%) vs. 71.4% (35.9%-91.8%)	26.0 (23.5–27.8) vs. 50.0 (45.5–59.5)	0.018
Operator experience (expert vs. trainee)	80.0% (49.0%-94.3%) vs. 66.7% (20.8%-93.9%)	30.5 (25.5–47.8) vs. 58.0 (54.5–59.5)	0.042
Catheter type (movable vs. non-movable)	66.7% (30.0%-90.3%) vs. 85.7% (48.7%-97.4%)	50.5 (29.8–56.3) vs. 33.0 (27.5–45.5)	0.720
SEMS, self-expanding metal stent.			

### Scope tip diameter-based analysis: ≤ 10 mm vs. > 10 mm

To evaluate the impact of scope tip diameter on procedure outcomes, we categorized scopes into those with a distal tip diameter ≤ 10 mm (JF-260V, pCF-H290TI, pCF-H290TI→JF-260V) and > 10 mm (CF-H260AI, CF-HQ290I). The technical success rate was 83.3% (5/6; 95% CI 43.6%-97.0%) in the ≤ 10-mm group compared with 71.4% (5/7; 95% CI 35.9%-91.8%) in the > 10-mm group ( *P* = 1.000). Median procedure time was 29.0 minutes (IQR 23.5–45.8) in the ≤ 10-mm group and 50.0 minutes (IQR 34.5–59.5) in the > 10-mm group ( *P* = 0.115).

### Hood-based analysis: tapering vs. non-tapering

When comparing tapering hoods (e.g., ST-short, Cast) to non-tapering hoods (e.g., attachment, ST-CB1), technical success rates were similar (83.3%; 95% CI 43.6–97.0% vs. 71.4%; 95% CI 35.9%-91.8%; *P* = 1.000). Median procedure time was 26.0 minutes (IQR 23.5–27.8) in the tapering hood group and 50.0 minutes (IQR 45.5–59.5) in the non-tapering hood group ( *P* = 0.018).

### Operator experience-based analysis: expert vs. trainee

Expert operators achieved a technical success rate of 80.0% (8/10; 95% CI 49.0%-94.3%), whereas trainees had a success rate of 66.7% (2/3; 95% CI 20.8%-93.9%; *P* = 1.000). Median procedure time was 30.5 minutes (IQR 25.5–47.8) in the expert group and 58.0 minutes (IQR 54.5–59.5) in the trainee group ( *P* = 0.042).

### Catheter-based analysis: movable vs. non-movable tip

When categorized based on the initially selected catheter, the technical success rate was 66.7% (4/6; 95% CI 30.0%-90.3%) in the movable-tip catheter group and 85.7% (6/7; 95% CI 48.7%-97.4%) in the non-movable-tip catheter group (p = 0.559). The median procedure time was 50.5 minutes (IQR 29.8–56.3) in the movable-tip catheter group and 33.0 minutes (IQR 27.5–45.5) in the non-movable-tip catheter group ( *P* = 0.720).

## Discussion

This study investigated the technical success rate and procedure efficiency of SEMS placement in the right colon, highlighting the particular difficulty of placement in the ileocecal region. Our results demonstrated that the technical success rate in the ileocecal region was 76.9% (95% CI 49.7%-91.8%), which was significantly lower than that in the other right colonic segments (98.3%, 95% CI 91.0%-99.7%) ( *P* = 0.017). This difference highlights the greater anatomical and technical complexity inherent in SEMS placement in the ileocecal area, underscoring the necessity for specialized procedure strategies tailored specifically to this challenging anatomical location.

Previous studies have reported significantly longer procedure times for ileocecal SEMS placement 9 ; however, our study did not find a significant difference in treatment time (ileocecal: median 40.0 min [IQR 30.0–56.0] vs. other right colonic segments: median 35.0 min [IQR 26.0–52.0], *P* = 0.934). This discrepancy may be attributed to the pre-procedure strategies and device selection optimizations implemented in our study. In particular, we routinely employed a contrast enema prior to the procedure—a method we defined as the CLEAN approach. This technique not only provided a laxative effect 10 but also offered valuable anatomical insights such as stricture length, angulation, and residual fecal load, which informed selection of procedure devices. For instance, when the enema revealed a sharp angulation, a more flexible scope or a tapered transparent hood was preferable. Conversely, in cases with straight strictures and heavy stool burden, a larger-caliber scope with greater suction capacity and a conventional hood was selected. These individualized adjustments likely contributed to maintaining procedure efficiency despite the anatomical complexity of the ileocecal region.

Moreover, in this study, no complications were observed among the ileocecal cases. This may be attributable to the specific types of stents utilized. Only low-axial-force stents (Natur fit and Niti-S) were used for SEMS placement in the ileocecal region, whereas high-axial-force stents such as WallFlex were not utilized. This may have contributed to lack of major complications such as perforation and bleeding 11 12 .

### Subgroup analysis interpretation

In this study, we evaluated the impact of catheter tip design (movable-tip vs. non-movable-tip) on technical success rates and procedure times. Although no statistically significant difference was observed between the two catheter types, clinical observations suggest potential advantages for movable-tip catheters. Notably, in Case 3, an initial attempt using a non-movable catheter (TandemXL) failed to traverse the stricture, but switching to a movable-tip catheter (PR-233Q) enabled successful guidewire passage and stent placement. Similar findings have been reported in previous studies, which highlighted improved guidewire maneuverability and successful stricture traversal with movable-tip catheters, such as the Swing Tip Cannula 13 14 , and with ERCP catheters featuring flexible tips in challenging ileocecal obstructions 15 . Thus, despite absence of statistical significance, the clinical utility of movable-tip catheters in anatomically complex cases warrants further investigation.

In addition, the shape of the endoscopic hood appeared to influence procedure efficiency. Although the technical success rate was comparable, procedure time was shorter in cases in which a tapered hood was used (median 26.0 min [IQR 23.5–27.8] vs. median 50.0 min [IQR 45.5–59.5], *P* = 0.018), suggesting that tapered hoods may facilitate visualization and device manipulation, particularly in the ileocecal region where endoscopic stability is critical. This finding aligns with a previous study that demonstrated the benefits of a small-caliber tapered transparent hood, which improved endoscopic visibility and guidewire insertion by limiting blood inflow and providing a more controlled visual field during colorectal self-expanding metal stent placement 16 .

Procedure experience has been previously reported to correlate significantly with clinical outcomes in colorectal SEMS placement. Although the optimal threshold of experience varies across different studies, accumulating evidence suggests that a minimum experience of 20 to 30 procedures is required to achieve consistent improvement in technical success rates 17 18 . Accordingly, in the present study, we defined "expert" as an operator who had previously performed at least 20 SEMS placements. Operator experience significantly influenced procedure efficiency: expert endoscopists completed SEMS placement in a median of 30.5 minutes (IQR 25.5–47.8), compared with 58.0 minutes (IQR 54.5–59.5) for trainees ( *P* = 0.042). This notable difference underscores the critical importance of technical proficiency when managing the anatomical complexity specific to the ileocecal region. Our findings are consistent with those of Small et al., who similarly demonstrated that increased operator experience was associated with higher procedure success rates and lower complication risks in SEMS placement for malignant colonic obstruction, reinforcing the necessity for advanced endoscopic expertise in optimizing clinical outcomes 19 .

This study evaluated the impact of endoscope tip diameter on SEMS placement in the ileocecal region, categorizing the scopes into ≤ 10 mm (JF-260V, pCF-H290TI, or pCF-H290TI→JF-260V) and > 10 mm (CF-H260AI or CF-HQ290I). Technical success rates were 83.3% (5/6) in the ≤ 10-mm group and 71.4% (5/7) in the > 10-mm group ( *P* = 1.000), showing no statistically significant difference. Procedure efficiency, evaluated by median procedure time, also did not differ significantly between groups (≤ 10 mm: median 29.0 min [IQR 23.5–45.8]; > 10 mm: median 50.0 min [IQR 34.5–59.5]; *P* = 0.115).

Although smaller-diameter scopes typically offer enhanced flexibility due to a smaller curvature radius—which may facilitate maneuverability through tortuous anatomy such as the ileocecal region—our data did not statistically confirm improved procedure efficiency or success rates. Conversely, larger-diameter scopes provide superior suction capabilities, which may help maintain clearer visualization and remove fecal residue. In addition, a side-viewing endoscope (JF-260V) was used in one case in which guidewire passage was particularly difficult with a forward-viewing endoscope due to severe angulation at the stricture site. However, with JF-260V, the guidewire was successfully advanced, facilitating SEMS placement 20 . This suggests that side-viewing endoscopes may offer advantages in select cases in which conventional approaches fail. Given the limited sample size and absence of statistical significance, these observations remain hypothesis-generating, and further investigation with larger cohorts is necessary. Further studies are required to establish clearer criteria for optimal scope selection based on anatomical and procedural considerations.

### Analysis of failed cases

In our study, two failed cases were primarily due to inability to pass the guidewire through the stricture. In Cases 9 and 12, even with use of a movable-tip catheter, the strictures could not be traversed. Because guidewire manipulation is known to be a major cause of perforation during SEMS placement 21 , gentle and cautious manipulation remains critical. When encountering significant difficulty, clinicians should consider early discontinuation of the procedure or an alternative therapeutic strategy, including surgical intervention, to avoid complications. In addition, one instance of stent jumping observed in our study might have been associated with the incremental "cell-by-cell" expansion characteristic of the Natur fit stent, which may result in unexpected retraction during deployment. Operators should carefully consider stent characteristics and deployment methods when managing complex ileocecal strictures. These findings remain hypothesis-generating, and future studies with larger cohorts are warranted to validate optimal management strategies in such challenging clinical scenarios.

### Limitations

Several limitations should be acknowledged in our study. First, given the retrospective design, inherent selection bias, incomplete data, and non-standardized procedure interventions might have affected the outcomes. In addition, the relatively small sample size in the ileocecal group could limit the statistical power, necessitating careful interpretation of the findings. These issues underscore that our results should be viewed as preliminary and hypothesis-generating rather than conclusive. Detailed exploration of prospective research directions, including large-scale and randomized controlled studies, is discussed further in the Future Directions section.

### Future directions

Although this study highlights the challenges associated with SEMS placement in the ileocecal region, the limited sample size necessitates further large-scale investigations. Future research should aim to enhance robustness of statistical analyses through multicenter collaborative studies, thereby increasing sample size and improving statistical power. In addition, a more comprehensive evaluation of long-term outcomes of SEMS placement, including patency rates and late complications, is warranted. Given the technical difficulties observed in this study, further investigations should explore whether newly designed low-axial-force stents or flexible catheters can improve success rates in the ileocecal region. Moreover, in cases in which standard forward-viewing colonoscopes fail, potential benefits of side-viewing endoscopes should be assessed to determine their role in overcoming anatomical challenges.

## Conclusions

This study demonstrated that SEMS placement in the ileocecal region is significantly more challenging than in other parts of the right colon due to its unique anatomical complexity. Although movable-tip catheters and tapered hoods showed potential clinical benefits, further research with larger cohorts is necessary to validate their effectiveness definitively. Exclusive use of low-axial-force stents likely contributed to absence of major complications, although careful consideration of their deployment characteristics remains important. Future studies should focus on clearly defining optimal strategies, including device selection, hood design, and operator training, to improve SEMS placement outcomes specifically in the anatomically challenging ileocecal region.
